# Environmental factors associated with overweight among adults in Nigeria

**DOI:** 10.1186/1479-5868-9-32

**Published:** 2012-03-27

**Authors:** Adewale L Oyeyemi, Babatunde O Adegoke, Adetoyeje Y Oyeyemi, Benedicte Deforche, Ilse De Bourdeaudhuij, James F Sallis

**Affiliations:** 1 Department of Physiotherapy, College of Medical Sciences, University of Maiduguri, Maiduguri, Nigeria; 2 Department of Physiotherapy, College of Medical Sciences, University of Ibadan, Ibadan, Nigeria; 3 Department of Biometry and Biomechanics, Faculty of Physical Education and Physiotherapy, Vrije Universiteit Brussels, Brussels, Belgium; 4 Department of Movement and Sports Sciences, Faculty of Medicine and Health Sciences, Ghent University, Ghent, Belgium; 5 Department of Family & Preventive Medicine, University of California, San Diego, USA

**Keywords:** Overweight, Obesity, Neighborhood environment, Africa.

## Abstract

**Background:**

Understanding environmental factors related to obesity can inform interventions for the world wide obesity epidemic, yet no study has been conducted in this context in Africa. This study examined associations between neighbourhood environment variables and overweight in Nigerian adults.

**Methods:**

A total of 1818 randomly selected residents (age: 20-65 years, 40% female, 31% overweight and 61.2% response) living in high and low socioeconomic (SES) neighbourhoods in Metropolitan Maiduguri, Nigeria, participated in a cross-sectional study. Anthropometric measurements of height and weight and an interview-assisted self-reported measure of 16 items of perceived neighborhood environments were conducted. The primary outcome was overweight (body mass index [BMI] > or = 25 kg/m^2^) vs. normal weight (BMI = 18.5-24.9 kg/m^2^).

**Results:**

After adjustment for sociodemographic variables, overweight was associated with distant access to commercial facilities (odds ratio [OR], 1.49; 95% confidence interval [CI], 1.02- 2.18), poor neighbourhood aesthetics (OR, 1.58; 95% CI, 1.16-2.09), perceiving garbage and offensive odours in the neighbourhood (OR, 1.41; 95% CI, 1.05-1.89) and feeling unsafe from crime at night (OR, 1.47; 95% CI, 1.13- 1.91) and unsafe from traffic (OR, 1.56; 95% CI, 1.17-2.07) in the total sample. Significant interactions regarding overweight were found between gender and four environmental variables, with low residential density (OR, 1.39; 95% CI, 1.02-1.93) and poorly maintained pedestrian pathways (OR, 1.89; 95% CI, 1.13-3.17) associated with overweight in men only, and absence of beautiful things (OR, 2.23; 95% CI, 1.42-3.50) and high traffic making it unsafe to walk (OR, 2.39; 95% CI, 1.49-3.83) associated with overweight in women only. There were few significant interactions between environmental factors and neighborhood SES regarding overweight.

**Conclusion:**

Neighbourhood environment factors were associated with being overweight among Nigerian adults. These findings support previous reports in international literature, but should be replicated in other African studies before any firm conclusions can be drawn.

## Background

Overweight and obesity (ie body mass index (BMI) > 25 kg/m^2^) are becoming increasingly prevalent worldwide [1-4], and have been associated with adverse health conditions such as hypertension, coronary heart diseases, diabetes, certain forms of cancer and reduced quality of life [1,5-7]. The problem of obesity is also rapidly increasing in developing countries with more than 115 million people suffering from obesity related problems [8]. The trend in the rising prevalence of obesity and related morbidity and mortality in developing countries has been attributed to rapid urbanization, nutrition transition and reduced physical activity [1,9,10]. Prevalence of obesity ranges between 3.3% and 18.0% in sub-Saharan African countries and has become a leading risk factor for cardiovascular diseases (CVD) and diabetes in urban areas of Africa [11]. Chronic diseases which include CVD, stroke, type-2 diabetes, cancer and obesity will by 2020 account for almost three quarters of all deaths worldwide, with 71% of deaths from CVD and 70% of deaths from diabetes projected to occur in developing countries [1,9].

Given the current obesity epidemic and its projected consequences, identifying effective population based interventions has become a public health priority in sub-Saharan Africa [11,12]. The problem of obesity is multifactorial, and prevention of weight gain can theoretically be achieved by altering the imbalance between energy consumed and expended [1,13]. However, complex behavioural and social factors including environments that promote unhealthy food choices and discourage physical activity are thought to be contributing to the imbalance driving the epidemic of population wide obesity [14-17]. The ecological models based on the principle that health behaviours are influenced by multiple levels, indicate that the environment has a significant effect on diet, physical activity and obesity [18,19]. These models have been recommended as a framework for studying health behaviours [20], because they can guide interventions that affect large populations over a long period of time [21]. In this context, the associations between neighbourhood environmental factors and physical activity have been reported in many countries across the continents [22,23], but no study has examined the independent association of environmental variables with overweight and obesity in African countries.

Specific neighbourhood environmental factors and urban sprawl have been associated with overweight and obesity among adults [24-29], and lack of pedestrian amenities, difficult-to-access destinations, poorly-connected street patterns, and neighbourhood safety perceptions have all been hypothesized to contribute to reduced physical activity and development of obesity [30-32]. However, many of these previous studies have been conducted in a few western developed nations, and findings may not apply in the developing countries. To date, research focusing on the relation of the built environment to overweight and obesity has rarely been conducted anywhere in Africa.

Africa-specific studies on the environment-overweight association are needed to guide evidence and develop effective population based interventions that can be tailored to the unique African context. Understanding the environmental factors associated with overweight and obesity among African adults can be critical for establishing the environmental approach as a viable international strategy for obesity control, and could provide clues about how the worldwide epidemic of obesity can be prevented before it becomes strongly established in African countries. The objective of this study was therefore to examine the independent associations of neighbourhood physical activity related environmental variables with overweight among Nigerian adults. This study used self-reported attributes of the neighbourhood environment rather than the objective approach of measuring access to stores, green spaces and food environments.

## Methods

### Participants and procedure

In this cross-sectional study, participants were systematically recruited from 38 neighbourhoods categorized into high and low socioeconomic status (SES) by the ministry of urban planning and development in Maiduguri, Nigeria. Maiduguri is the largest and the capital city in Borno State, North Eastern Nigeria, with an estimated population of 4,151,193 people. According to the 2003 Nigerian Demographic and Health Survey [33], high SES areas are mostly inhabited by people who are gainfully employed (elites), have more than secondary school education, and composed of many houses with functional potable water sources and modern sanitary facilities (flush toilet). Low SES areas mostly have residents who are self- employed (artisans, traders etc.) or unemployed, with varying educational qualifications, and composed of few houses with functional water sources and modern sanitary facilities. For the 1991 population census in Nigeria, a neighbourhood was defined as one or more enumeration areas with a minimum of 50 households [33].

Eleven trained interviewers and the principal investigator recruited participants using a door-to-door strategy. In each neighbourhood, households were enumerated on site and every odd-numbered household was approached for study interest and eligibility. When no one was at home or an eligible adult resided in the household but was not available, the interviewers made a maximum of five return visits for recruitment purposes. Participants who met the eligibility criteria of (1) living within the identified neighbourhoods in the last year, (2) being 20 to 65 years, (3) willing to provide objective weight and height measurements, (4) not being underweight, and (5) being able to complete or understand surveys in either English or Hausa languages were invited to participate in the study.

After recruitment, participants were introduced to the study and their informed consent was obtained. Height and weight was measured using standardized procedures and the questionnaire was self- administered with research staff in attendance to assist participants who had questions about the survey. Among the 2970 individuals contacted for the study, 612 (20.6%) refused participation, 301 (10.1%) were not eligible, and 2057 (69.3%) agreed to participate. Among the individuals who agreed to participate, 1818 (88.4%) provided complete and usable survey information. Age and being underweight were the primary reasons for ineligibility. Response rate was 61.2% (n = 1818/2970). All participants provided informed consent, and the study was approved by Human Research Ethic Committee of the University of Maiduguri Teaching Hospital, Nigeria. Data were collected from August 2010 to September 2011.

### Measures

#### Anthropometry

Body height and body weight was determined by standard anthropometric methods. Height was measured to the nearest 0.1 cm in bare feet with participants standing upright against a Holtain portable stadiometer (Crymych, United Kingdom). Weight was measured to the nearest kilogram, with participants lightly dressed using a portable bathroom weighing scale calibrated from 0-120 kg. BMI was calculated as weight (kg) divided by the square of the height (m^2^). The World Health Organization (WHO) principal cutoff points for BMI were used to create the categories: underweight (< 18.5 kg/m^2^), normal weight (18.5- < 25 kg/m^2^), overweight (25- < 30 kg/m^2^) and obese (> 30 kg/m^2^) [1]. For present analyses, participants were categorized as normal weight group and overweight group (defined as BMI over 25, including BMI > 30 kg/m^2^).

#### Environmental assessment

An adapted self-administered version of the Physical Activity Neighbourhood Environment Scale (PANES) was used to assess participants' perception of neighbourhood environmental factors. The principal investigator and an expert group (local and international) composed of public health scientists, geographers, town and urban planners, and housing and transportation executives adapted the PANES for use in Nigeria. The 17-item PANES was originally designed by the International Physical Activity Prevalence Study group for brief assessment of variables believed to be related to the activity-friendliness of neighbourhoods [34]. Neighbourhood environmental variables assessed by the adapted PANES included (1) residential density (one question), (2) access to destinations (three questions), (3) connectivity of the street network (one question), (4) infrastructures for physical activity and walking (three items), (4) social environment (one question), (5) aesthetics (three questions), and (6) neighborhood safety (four questions). One item on household motor vehicles was removed from the PANES during the adaptation procedure. With the exception of the question on residential density, all items were phrased as statements about attributes of the neighbourhood, with the following response options: strongly disagree, somewhat disagree, somewhat agree, strongly agree, don't know/not sure or doesn't apply response. For the item on residential density, participants were asked if they perceived their neighbourhood to have many apartments of shared facilities and multiple households or flats of 3 or more stories (high residential density). For the purpose of statistical analysis, a dichotomous variable was constructed. Responses to items were collapsed into two categories: "agree" (strongly agree and somewhat agree) and "disagree" (strongly disagree and somewhat disagree). The PANES has been previously used among Nigerian young adults and showed good reliability [35], comparable with reliability findings among adults in the United States [36] and Sweden [37].

#### Sociodemographic characteristics

Information on age, gender, marital status, religion, income, educational level and employment status was elicited from the participants. Marital status was classified as married/ever been married and not married. Educational level was classified as more than secondary school education, secondary school education, and less than secondary school education. Employment status was classified into employed (government/private and self- employed) and unemployed (homemaker, student, retired, or unable to work). Income was categorized into 4 groupings based on NAIRA/month (15 000 NAIRA ≈ 100 US Dollars).

#### Statistical analysis

Descriptive statistics were computed for all variables. The independent association of perceived environmental variables with overweight as the dependent variable was examined using logistic regression analysis. Adjusted odd ratios (ORs) and 95% confidence intervals (CI) were calculated for each environmental variable. Interactions between environmental factors and gender regarding overweight were explored by including interaction terms in the regression model. In addition, the interactions of neighborhood SES with environmental variables for overweight were explored, and in case of significant interactions, separate logistic regression analyses were performed in both high and low SES neighborhood. Regression analyses were adjusted for sociodemographic variables (age, gender, neighbourhood location, employment status, and educational level). Income was not included in the regression models due to large number of missing responses (n = 135) and its significant correlation with employment status (ρ = 0.35, p < 0.01) and educational level (ρ = 0.51, p < 0.01). All statistical analyses were conducted using SPSS version 15 (SPSS. Inc., Chicago, IL).

## Results

A total of 1818 participants comprising 39.9% women and 60.1% men, with mean age of 32.3 ± 10.0 years and BMI of 24.0 ± 4.0 kg/m^2^, participated in this study. About 31.0% of the participants were overweight (22.8% with BMI > 25 kg/m^2^; 8.1% with BMI > 30 kg/m^2^). Compared with normal weight participants, overweight individuals were more likely to be older, women, married or been married, have at least secondary school education, employed and of lower income status (Table 1).

**Table 1:** Socio-demographic characteristics and physical activity level of all participants and according to body mass status

Characteristics	Total Sample		Normal weight		Overweight/Obese		
		
	N = 1818		N = 1255 (69.0%)		N = 563 (31.0%)		*p*
	**Mean (± SD)**		**Mean (± SD)**		**Mean (± SD)**		
**Age (years)**	32.3 ± 10.0		31.1 ± 9.9		35.4 ± 9.9		< 0.001†
**Height (m)**	1.67 ± 0.07		1.67 ± 0.1		1.66 ± 0.1		0.070†
**Weight (kg)**	66.7 ± 11.7		61.3 ± 7.1		78.7 ± 10.9		< 0.001†
**BMI (Kgm^-2^)**	24.0 ± 4.0		21.8 ± 1.8		28.8 ± 3.4		< 0.001†
	**N**	**%**	**N**	**%**	**N**	**%**	
**BMI Prevalence**							< 0.001††
Normal weight	1255	69.0	1255	100	0.0	0.0	
Overweight	415	22.8	0	0.0	415	73.7	
Obese	148	8.1	0	0.0	148	26.3	
**Gender**							
Female	725	39.9	480	38.3	245	43.4	0.047†
Male	1093	60.1	774	61.7	319	56.6	
**Marital Status**							
Married	1044	57.4	665	53.1	428	75.9	< 0.001††
Single	774	42.6	589	47.0	136	24.1	
**Ethnic group**							
Hausa/Fulani	534	29.4	376	30.0	158	28.0	< 0.001††
Kanui/Shuwa	619	34.0	485	38.7	134	23.8	
Others	665	36.6	429	31.3	272	48.2	
**Educational level**							
> Secondary School	533	29.3	325	25.9	208	36.9	< 0.001††
Secondary School	736	40.5	522	41.6	214	37.9	
< Secondary School	549	30.2	407	32.5	142	25.2	
**Employment status**							
Employed	1077	59.2	726	57.9	351	62.2	0.043††
Unemployed	741	40.8	528	42.1	213	37.7	
Monthly Income (Naira)*							
< 15 000	607	36.1	454	38.7	153	30.1	< 0.001††
16 000- 45 000	650	38.6	466	39.7	184	36.1	
46 000- 90 000	247	14.7	159	13.5	88	17.3	
> 90 000	179	10.6	95	8.1	84	16.5	

SD- Standard Deviation

BMI- Body Mass Index

*- Do not add to 1818 due to missing values

† - p-values based on independent *t*-test statistic;

†† - p value based on Chi-Square statistic

A lower proportion of overweight adults compared with their normal weight counterparts perceived their neighbourhood as having high residential density (32.3% vs 38.1%), many non-residential places such as schools, workplaces and hospitals (83.7% vs 88.8%), proximal access to public transport (79.0% vs 83.9%) and being safe to bicycle (76.6% vs 85.1%). In addition, a higher proportion of overweight adults compared to normal weight participants perceived their neighbourhood as unsafe from traffic to walk (30.3% vs 25.1%). (Not shown in table).

Table 2 showed the independent associations of each neighbourhood environment variable with overweight. After adjustment for sociodemographics, five out of 16 neighbourhood environment variables were significantly associated with overweight. Participants who did not report commercial places such as shops, stores and markets to be within walking distance of their homes were 49% more likely to be overweight than those who reported proximal facilities (OR = 1.49; CI = 1.02- 2.18). Participants who did not perceive many beautiful things in their neighbourhood were 58% more likely to be overweight than those who reported many beautiful things (OR = 1.58; CI = 1.16- 2.09). Similarly, those who perceived their neighbourhood not to be free from garbage and offensive odour were 41% more likely to be overweight than those who perceived high level of garbage and offensive odour (OR = 1.41; CI = 1.05- 1.89). Participants who perceived their neighbourhood as unsafe from crime during the night were 47% more likely to be overweight (OR = 1.47; CI = 1.13- 1.91), and those who perceived their neighbourhood as unsafe from traffic to walk were 56% more likely to be overweight (OR = 1.56; CI = 1.17- 2.07) than those who perceived their neighbourhood as safe.

**Table 2:** Association between perceived neighborhood environment factors and overweight

			Overweight N = 564 (31.0%)
	
	N	%	Adjusted OR (95% C.I)†
**Residential density**			
Low	382	67.7	1.06 (0.83- 1.37)
High	182	32.3	1.00
**Access to commercial places**			
Disagree	189	33.5	1.49 (1.02- 2.18)*
Agree	375	66.5	1.00
**Access to non-residential places**			
Disagree	92	16.3	1.02 (0.74- 1.39)
Agree	472	83.7	1.00
**Access to public transport**			
Disagree	119	21.0	1.01 (0.74- 1.40)
Agree	445	79.0	1.00
**Presence of recreational centers**			
Disagree	159	28.2	0.96 (0.68- 1.35)
Agree	405	71.8	1.00
**Presence of pedestrian pathways**			
Disagree	167	29.6	1.17 (0.85- 1.62)
Agree	397	70.4	1.00
**Maintenance of pathways**			
Poor	93	16.5	1.18 (0.77- 1.79)
Good	471	83.5	1.00
**Presence of beautiful things**			
Disagree	230	40.8	1.58 (1.16- 2.09)*
Agree	334	59.2	1.00
**Absence of unattended animals**			
Disagree	337	67.0	1.19 (0.89- 1.59)
Agree	186	33.0	1.00
**Absence of garbages and foul odors**			
Disagree	259	45.9	1.41 (1.05- 1.89)*
Agree	305	54.1	1.00
**Seeing people active**			
Disagree	79	14.0	0.75 (0.52- 1.09)
Agree	485	86.0	1.00
**Connectivity of street**			
Poor	82	14.5	0.78 (0.55- 1.10)
Good	482	85.5	1.00
**Traffic safety for bicycling**			
Not safe	132	23.4	1.29 (0.90- 1.87)
Safe	432	76.6	1.00
**Traffic safety for walking**			
Not safe	171	30.3	1.56 (1.17- 2.07)*
Safe	393	69.7	1.00
**Crime Safety during the day**			
Not safe	114	20.2	0.91 (0.66- 1.27)
Safe	450	79.8	1.00
**Crime safety at night**			
Not safe	245	43.3	1.47 (1.13- 1.91)*
Safe	319	56.6	1.00

CI indicates confidence intervals; and OR, odds ratio

† Adjusted for age, gender, neighborhood location, marital status, ethnic group, employment status, and educational level

* P < 0.05

Significant interactions regarding overweight were found between gender and four of the environmental factors (Table 3). While men would be more likely to be overweight when they perceived low residential density (OR = 1.39, CI = 1.02- 1.93) and poorly maintained pedestrian pathways (OR = 1.89, CI = 1.13- 3.17), these environmental factors were not related with overweight in women. On the other hand, perceiving no beautiful things in the neigborhood (OR = 2.23, CI = 1.42- 3.50) and high traffic making it unsafe to walk (OR = 2.39, CI = 1.49- 3.83) were related with higher levels of overweight in women but not in men. (Not shown in table).

**Table 3:** Significance of interactions between environmental variables, and gender and neighborhood socioeconomic status by binary logistic model

		Overweight		
		
	Interaction terms with gender and neighborhood SES			
	
	Gender		Neighborhood SES	
	
	OR†	(95% C.I)	OR†	(95% C.I)
**Residential density (low)**	**1.71**	(1.02- 2.86)*	1.07	(0.37- 3.12)
**Access to commercial places (Disagree)**	1.42	(0.74- 2.74)	1.25	(0.61- 2.58)
**Access to non-residential places (Disagree)**	1.35	(0.59- 3.04)	0.67	(0.28- 1.57)
**Access to public transport (Disagree)**	0.95	(0.49- 1.85)	0.72	(0.35- 1.51)
**Presence of recreational centers (Disagree)**	0.67	(0.33- 1.37)	1.98	(0.86- 4.55)
**Presence of pedestrian pathways (Disagree)**	0.89	(0.46- 1.75)	1.98	(0.92- 4.05)
**Maintenance of pathways (Poor)**	**3.08**	(1.26- 7.49)*	1.72	(0.54- 5.49)
**Presence of beautiful things (Disagree)**	**1.81**	(1.05- 3.13)*	**1.29**	(1.02- 2.86)*
**Absence of unattended animals (Disagree)**	1.23	(0.68- 2.25)	0.89	(0.49- 1.65)
**Absence of garbages and foul odors (Disagree)**	0.92	(0.49- 1.71)	2.12	(0.83- 5.39)
**Seeing people active (Disagree)**	0.74	(0.34- 1.58)	0.54	(0.19- 1.46)
**Connectivity of street (Poor)**	1.79	(0.88- 3.65)	0.78	(0.42- 1.83)
**Traffic safety for bicycling (Not safe)**	1.13	(0.51- 2.55)	0.99	(0.45- 2.20)
**Traffic safety for walking **(**Not safe)**	**2.19**	(1.22- 3.93)*	1.66	(0.87- 2.71)
**Crime Safety during the day **(**Not safe)**	1.08	0.55- 2.12)	0.66	(0.24- 1.83)
**Crime safety at night **(**Not safe**)	1.18	(0.69- 2.04)	**2.35**	(1.66- 4.24)*

OR_ odds ratio' *_ statistically significant (*p *< 0.05)

†_ Adjusted for age, neighborhood location, marital status, ethnic group, employment status, and education

†_ Adjusted for age, gender, marital status, ethnic group, employment status, and education

Furthermore, significant interactions for overweight were only found between neighborhood SES and two environmental factors as they related to overweight (Table 3). Perception of poor aesthetics was significantly related with higher levels of overweight in adults who reside in low SES Neighborhoods (OR = 1.35, CI = 1.02- 1.81) but not related to overweight in adults who reside in high SES neighborhoods. However, perception of high crime rate at night making it unsafe to walk was significantly related to higher levels of overweight in adults who resided in high SES neighbourhoods (OR = 2.36, CI = 1.20- 4.64) but not related to overweight in adults who resided in low SES neighbourhoods. (Not shown in table).

## Discussion

The major finding in the present study was that perceptions of five neighbourhood environment variables unfavorable to physical activity were associated with overweight among African adults. The effect sizes were substantial, with unsupportive environment variables associated with 40% to 60% higher risk of overweight. Present findings indicate that neighbourhood environments have robust associations with body mass index status among Nigerian adults. To our knowledge, no study has been published on how neighbourhood environment variables relate to overweight and obesity among adults in any African country.

Consistent with studies in the United States [28,38] and Australia [29], being overweight was associated with perception of commercial facilities such as shops, stores and markets not to be within proximal distance of homes. Because people engaged in more physical activity, particularly walking for transportation, when they perceived proximal rather than distant destinations [22,37], availability of facilities within walking distance can positively impact on energy expenditure which may assist in weight control. Locating commercial facilities within neighbourhoods, termed mixed land use, may therefore be a viable strategy for obesity prevention in Nigeria. The replication of this kind of finding in the developing African continent may also suggest an international relevance of land use-mix access as an important environmental variable that can be manipulated by policy for effective intervention to prevent overweight and obesity.

The environmental attribute associated with the highest likelihood of being overweight was absence of many beautiful things in the neighbourhood. This finding in addition to the association between overweight and perceptions of presence of garbage and offensive odours on most streets in the neighbourhood affirms conclusions from previous findings [27,28]. This may suggest an important area for possible interventions because neighbourhoods with favourable aesthetics may encourage individuals to be physically active outdoors to enjoy the scenery. Reviews have found neighbourhood aesthetics to be consistently related to recreational physical activity and walking [39-41]. Ensuring good aesthetic quality of the neighbourhood may be a practical and effective policy for encouraging physical activity and reducing overweight and obesity in African adults.

In this study, a higher proportion of overweight individuals compared to their normal weight counterparts tend to report much traffic in their neighbourhoods' street making it difficult to walk. Heavy traffic has previously been associated with obesity among rural [15] and urban [25] residents in the United States, and residents of a metropolitan city in Australia [29]. Interestingly, another environmental variable on safety; high crime rate at night, was also associated with being overweight in the present study. It can be presumed that individuals who perceived threat from crime would be less likely to come outdoors to be physically active, and perceptions of heavy traffic in addition to this, can further compromise the choice of an individual to engage in neighbourhood based physical activity. Making the environments safe from traffic and crime may be particularly important for obesity control, because how people use the environment is thought to be dependent on their perceptions of how safe they feel the environment is [42], and this perception can be closely related to health [43]. It should however be noted that the association of these safety variables with physical activity have been inconsistent as indicated by multiple review [39-41].

Because sex differences in the associations between environment and overweight or obesity have not been studied often, those observed in the present study are of particular interest. Significant interactions regarding overweight were found between gender and four of the environmental factors, supporting a conclusion that different environmental factors may be relevant for obesity prevention among African men and women. This suggests the preferences of both women and men need to be considered in the design of activity-supportive and health-promoting neighborhoods. Poor aesthetics and traffic were related to overweight in women only. While good neighborhood aesthetics have been associated with walking among African young women and men [23], high speed of traffic is an important barrier to physical activity that seem to be specific to women in Nigeria [44]. Plausibly, African women may not engage in physical activity when they perceive their neighborhood as aesthetically unpleasing and dangerous due to high traffic, making them more likely to be overweight.

Also worth noting is that low residential density and poorly maintained pedestrian facilities were not related to overweight in the full sample, but positively related to being overweight in men only. These findings suggest the importance of considering sex-specific environmental interventions for obesity control in Africa. For example, pedestrian facilities may not be important to physical activity behaviours of African women because they may want to avoid other users, especially males that are not related to them. In contrast, African men may prefer to engage in physical activity in densely populated neighbourhoods with pedestrian facilities because of the stimulating and role-model effects of being seen engaging in a health enhancing behaviour. In addition, we found few significant interactions between environmental factors and neighborhood SES regarding overweight, suggesting that associations found between overweight and environmental variables may be similarly important across the entire range of SES.

This study has some important limitations which should be considered when interpreting the findings. Perceived, as opposed to objective, measures of neighbourhood environments were used. Previous studies have reported limited agreement between perceived and objective measures of neighbourhood characteristics, but it is not known which is more important to overweight and obesity [28,29]. The study did not include measures of the food environment, which may be important in explaining obesity. Although adjustment was made for neighborhood SES in the analysis, and the interactions of neighborhood SES with environmental variables for overweight were explored, neighborhood SES variables may cofound the outcome variables in this study, making it difficult to determine the relative influence of neighborhood SES and environmental variables on our findings. In addition, participants were younger on the average and were recruited from a single city, which may compromise environmental variability and limit generalization of findings to other African countries. The cross-sectional design utilized does not allow for causal relationships to be determined. Prospective or quasi- experimental studies are needed to strengthen the evidence of causality in this field. However, given the sparse literature on environmental interventions to prevent and control obesity in Africa, correlation studies such as this can provide leads for researchers and practitioners in planning future work. Strengths of the present study included the objective assessment of BMI and the use of neighbourhood environmental items that were tailored to the African context. The sample was also selected to represent a large range of socioeconomic status similar to the general population in North Eastern Nigeria [33]. This study adds to a growing evidence base of environmental correlates of overweight and makes a unique contribution regarding the African population.

In conclusion, neighbourhood environment factors like access to commercial destinations, neighbourhood aesthetics, and safety from crime and traffic were associated with overweight among Nigerian Adults. Unfavorable attributes were associated with 40% to 60% increased risk of obesity, which is substantial. The results suggest that previous findings from developed countries may be applicable in Africa, and interventions focusing on neighbourhood environmental factors can be considered as an important public health agenda in Africa in the current battle against the obesity epidemic. However, given the preliminary nature of this study, it is important to replicate these findings in other African studies before any firm conclusions can be drawn.

## Competing interests

The authors declare that they have no competing interests.

## Authors' contributions

ALO conceived, designed and drafted the manuscript; was responsible for data acquisition, conducted the statistical analysis and interpretation of data. BOA and AYO contributed to study design and drafting of the manuscript. BD, IDB and JFS participated in the design of the study, interpretation of data and critically revised the drafted manuscript for important intellectual content. All authors read and approved the final manuscript.
